# Transitional Care Program in Reducing Acute Hospital Utilization in Singapore

**DOI:** 10.3390/healthcare12212144

**Published:** 2024-10-28

**Authors:** Chong Yau Ong, Jun Jie Angus Ng, Kar Kwan Sandra Joanne Ng, Pei Yoke Tay, Mui Hua Jean Lee

**Affiliations:** 1 Transitional Care Community Medicine, Population Health & Integrated Care, Sengkang General Hospital, Singapore 544886, Singapore; 2 Lee Kong Chian School of Medicine, Nanyang Technological University, Singapore 308232, Singapore; 3 Community Nursing, Sengkang General Hospital, Singapore 544886, Singapore

**Keywords:** care continuity, health program, home care, home visit, hospital admission, multimorbidity, transitional care

## Abstract

(1) Background: The evidence to support transitional care in reducing acute hospital utilization is variable. Despite changes in the healthcare landscape with a rapidly aging population, there is a lack of local and regional studies to evaluate the effectiveness of transitional home care programs. This study investigates whether a transitional home care program delivered by an acute tertiary hospital can reduce acute hospital utilization. (2) Methods: A pre-post design was used to evaluate the effectiveness of the program. A total of 2004 enrolments from 1679 unique patients that fulfilled the criteria of enrolment were included. The transitional care program is delivered through telephone follow-up and home visits. The Wilcoxon Signed-Rank Test was used to assess the differences between the three periods of baseline, enrolment, and post-enrolment. (3) Results: All 2004 enrolments were analyzed. The re-attendances at the emergency department reduced significantly by 31.2% and 71.9% during enrolment and post-enrolment (*p* < 0.001), respectively. Similarly, patients had a 38.7% and 76.2% reduction in hospital admissions during enrolment and post-enrolment (*p* < 0.001), respectively. For patients who were admitted, there was no significant difference in the length of stay between these groups (*p* = 0.23). (4) Conclusions: The transitional home care program can effectively reduce emergency department re-attendances and inpatient admissions. Not only was the total number of emergency department re-attendances reduced significantly, but the number of frequent re-attendances also dropped significantly. The outcomes were consistent during COVID-19 and post-pandemic phases. These findings can be used as a guide in program planning and future scalability.

## 1. Introduction

Transitional care is an important aspect of healthcare that focuses on the coordination and continuity of patient care as they move between different levels of healthcare and across care settings [1]. It involves a systematic and well-coordinated approach to ensure a smooth transition from hospital to home or from one healthcare facility to another. The primary goals of transitional care are to maintain continuity of care, improve patient outcomes, and reduce hospital utilization [2].

As the healthcare landscape in Singapore grapples with a rapidly aging population with fewer caregivers, acute public hospital bed shortages and insufficient intermediate and long-term care capacity, the importance of effective transitional care becomes increasingly evident [3,4]. Physicians and healthcare allies have posed the challenge of providing comprehensive care to older adults riddled with a host of chronic conditions amidst their complex social backgrounds. With an increased prevalence of multimorbidities, along with the increased sub-specialties of the healthcare landscape [5,6], the complexity of coordinating care management from different parts of the healthcare industry is further heightened [7,8]. Transitional care interventions aim to reduce healthcare utilization and improve patient outcomes [9].

Sengkang General Hospital’s (SKH’s) transitional care program (Hospital to Home) typically involves an interdisciplinary team of doctors, nurses, and allied health professionals such as medical social workers, speech therapists, occupational therapists, and physiotherapists [10,11]. The main point of contact during home visits and phone call follow-ups is an assigned senior staff nurse or nurse practitioner [12]. The patients are identified and enrolled through the inpatient referral process before being discharged and through the hospital’s predictive list from medical informatics. Following the first encounter, a first home visit is usually scheduled in between three to seven days. This concurs with findings that most older adults are at highest risk for poor discharge outcomes. The physician accompanies the nurse for home visits for more complex cases to assess and stabilize their medical conditions. A physiotherapist, occupational, or speech therapist may visit patients at home to assess their home environments and perform rehabilitation interventions with the patients and/or their caregivers if they have problems with mobility, Activities of Daily Living (ADL), speech, and/or swallowing.

The transitional care program conducted by our hospital is a non-specialty program delivered through a mixture of physical home visits and telephone and messaging follow-ups. The components of the intervention are presented in Table 1.

Similar transitional care programs implemented in hospitals in Singapore [11,13,14] and overseas [15,16,17] have shown significantly lower acute hospital utilization, reduced re-admission rates and length of stay. Despite evolving changes in the healthcare landscape with a rapidly aging population, there is still a paucity of up-to-date local and regional studies on the outcomes of transitional home care. This study aims to examine whether a transitional care program can still meet its purpose to reduce hospital utilization indicators by reducing emergency department (ED) re-attendances and inpatient re-admissions and shortening patients’ length of stay in an acute tertiary hospital by 60% each [11,18].

## 2. Materials and Methods

### 2.1. Study Design

This was a retrospective study of patients enrolled and cared for under the transitional care program between 1 August 2020 and 30 June 2023. Data were obtained through the hospital’s medical informatics. A pre-post study design was employed on patients enrolled in the transitional care program (intervention). Each patient’s historical data (during the baseline period) served as his/her own control [11].

The baseline period studied was defined as three months prior to enrolment (pre-enrolment period), and the follow-up period was defined as three months after discharge from the transitional care program (post-enrolment period). The number of ED visits and inpatient admissions for the pre-enrolment, during enrolment, and post-enrolment periods were recorded for each enrolment case.

### 2.2. Study Setting and Population

SKH is one of Singapore’s new public hospitals, which has 1000 beds. SKH is an acute tertiary hospital offering a comprehensive suite of specialist care, inpatient and rehabilitation services, and emergency services. Operating since August 2018, it serves mainly the population of northeast Singapore, which accounts for 11 to 12% of Singapore’s population.

The patients were enrolled either from the ED, inpatient wards, specialist outpatient clinics or from the community, which includes other hospitals or organizations in the community. As this is not a specialty-based program, such patients are included: outstanding medical and/or caregiver issues, frequent admitters (≥2 in 6 months or ≥3 in a year), and frequent falls (≥2 in a year). Institutional persons (residents of long-term care facilities), foreign workers (due to inability to use the funding system), and patients already enrolled in transitional care or palliative programs offered by other hospitals and hospices (program duplication) were excluded from the program.

### 2.3. Outcome Variables

The primary outcome of the study is the number of ED re-attendances during enrolment and post-enrolment periods. We hypothesized that the latter number would be smaller than the former number, attributing the decrease in ED attendance to the transitional care program. The secondary outcomes are inpatient admissions and length of stay during enrolment and post-enrolment periods. We do not distinguish between planned/elective or unplanned re-admissions.

### 2.4. Statistical Analysis

Descriptive statistics were computed for the study population. The main diagnosis for each enrolment was determined as the diagnosis of either the most recent ED visit or inpatient admission in the pre-enrolment period. The diagnoses were counted, and the ten most common diagnoses were recorded. Descriptive statistics were analyzed using Microsoft Excel version M365 (Microsoft Inc., Redmond, WA, USA).

A comparison of healthcare utilization measured by ED re-attendances, inpatient admissions, and length of stay between pre-enrolment, during enrolment and post-enrolment was calculated using the Wilcoxon Signed-Rank Test.

While it is a common practice to exclude patients who had died, we decided to include them as this would reflect more accurate outcomes given that the patients nearing their end of life are usually re-admitted more often as their disease worsens.

Notably, when analyzing the length of stay, enrolments that had avoided re-admission were excluded because their inclusion would have resulted in a shorter length of stay than the actual due to inclusions of enrolments with zero-day admissions.

### 2.5. Ethical Approval

This study has been assessed by SingHealth Centralized Institutions Review Board to not require an ethics board review (CIRB Number 2023-2369) (Approval date 22 July 2023). Consent from participants was therefore not obtained.

## 3. Results

### 3.1. Characteristics of Study Population

The demographics of the patient population in this study are illustrated in Table 2. During the study period spanning 35 months, a total of 1679 unique patients were recruited. These patients had a total of 2004 enrolments, as some were enrolled more than once. 

Most patients are older adults aged 61 years old and above. Their mean clinical frailty score was 5.6, which is between mildly frail and moderately frail. Their mean Barthel Index of Activity Daily Living was 12.0 (0 being totally dependent and 20 being independent).

The three most common diagnoses of patients enrolled in the transitional care program were fluid overload, urinary tract infection, and pneumonia.

### 3.2. Effect on Rates of ED Re-Attendances and Inpatient Re-Admissions

**Figure 1:**
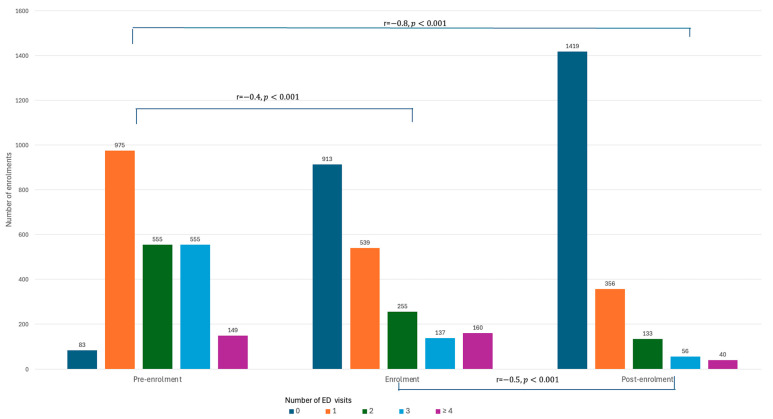
Comparison of ED visits during pre-enrolment, enrolment, and post-enrolment periods.

The number of ED re-attendances reduced significantly by 31.2% from pre-enrolment (n = 3508) to during enrolment (n = 2414) into the transitional care program. This reduction became more stark with a 71.9% reduction (n = 983) when measured post-enrolment (Supplementary Table S1). When categorized by the count of ED visits within the measured periods, the numbers of patients with one, two, three, and four or more ED visits dropped significantly during enrolment (r = −0.4, *p* < 0.001) and the post-enrolment period (r = −0.8, *p* < 0.001). This corresponded with the reciprocal rise in the number of those with no ED visits (Figure 1 and Supplementary Table S1).

**Figure 2:**
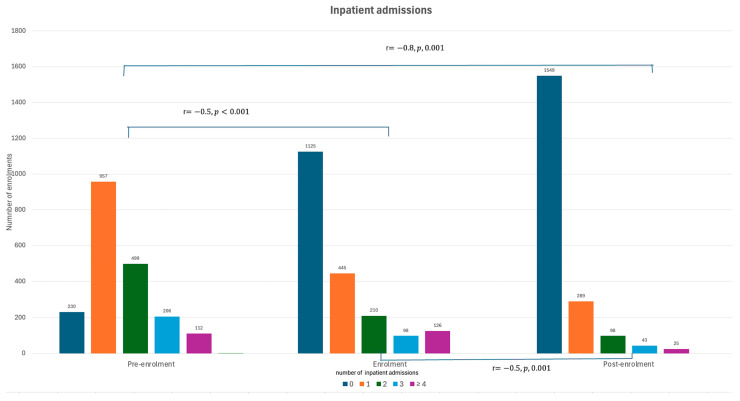
Comparison of inpatient admissions during pre-enrolment, enrolment, and post-enrolment periods.

Similarly, a reduction of 38.7% in inpatient admissions (n = 2414) was observed during enrolment into a transitional care program (from n = 3080). Compared to the pre-enrolment period, there was a reduction of 76.2% (n = 983) in inpatient admissions post-enrolment. The number of patients that had no inpatient admissions increased while those who were admitted one, two, three, and four or more times decreased significantly during (r = −0.5, *p* < 0.001) and after the transitional care program (r = −0.8, *p* < 0.001) (Figure 2 and Supplementary Table S2).

### 3.3. Effect on Length of Stay During Re-Admission

After excluding subjects that have no inpatient re-admissions, there was a non-significant, small decrease in the mean length of stay from the pre-enrolment (μ = 7.71 days) to the post-enrolment period (μ = 7.64 days) (r = −0.04, *p* = 0.230).

### 3.4. High Utilizers

We defined high utilizers as patients who had three or more ED visits during the 90-day pre-enrolment period. This is a stricter definition than the patient group, which is defined as high utilizers in another paper [19], which was patients with four or more ED visits in a full calendar year. This is based on our subgroup analysis of the former having at least double or triple the number of ED visits compared to the latter.

There were 391 enrolments that met the criteria. There was a significant reduction in ED visits among this group (r = −0.86, *p* < 0.001) during post-enrolment as compared to pre-enrolment (Supplementary Table S3). This reduction was already of a moderate effect during the enrolment period (r = −0.64, *p* < 0.001).

Similarly, the reduction in inpatient admissions for this group of patients was noted to be significant during the enrolment period (r = −0.61, *p* < 0.01) and further reduced post-enrolment (r = −0.86, *p* < 0.01).

### 3.5. Effect of COVID-19 on Outcome Variables

We compared two time periods of interest between 1 March 2021 to 25 April 2022 (421 days) and between 26 April 2022 to 30 June 2023 (431 days). The first period was when lockdown and movement restrictions affected Singapore. Although the restrictions were gradually relaxed, it was not until 26 April 2022 that the Disease Outbreak Response System Condition (DORSCON) level changed from Orange to Yellow, which signaled a significant easing of community measures.

The number of unique patients managed during the two periods was 511 and 643, respectively. The effect sizes of transitional care programs (pre-enrolment and post-enrolment) between the two periods were comparable (r = −0.7, *p* < 0.001 vs. r = −0.8, *p* < 0.001).

## 4. Discussion

### 4.1. Effect on Rates of ED Re-Attendances and Inpatient Re-Admissions

To our knowledge, this is the largest single-center study on transitional home care in Asia. Our study validates that the transitional care delivered by an acute tertiary hospital can effectively reduce the rates of ED re-attendances as well as inpatient re-admissions. This decrement was most significantly observed between the three-month pre-enrolment period and the three-month post-enrolment period. This is supported by current literature from similar care programs from other institutions, both in Singapore and abroad [11,13,14,15,16,17]. A meta-analysis and systematic review of transitional care programs generally yielded mixed results due to high heterogeneity in the population, intervention (delivery methods), and different time points of measured outcomes [17,20].

In contrast to some studies wherein the most significant impact was seen within the first 30 days after discharge [17] and plateaued or had a rebound effect measured towards the tail of enrolment [11,21], our study showed a further reduction in measured outcomes even at three months post-enrolment. This is in keeping with findings that transitional care is effective in reducing all-cause intermediate (31 to 180 days) to long-term re-admissions (181 to 365 days) [22]. This suggests that the knowledge and support imparted to the patient and their caregivers were still retained, and they were able to empower them for self-care even after being discharged by the transitional care team [10,16,23].

### 4.2. Effect on Length of Stay During Re-Admission

While our study showed a decrease in the length of stay in the hospital during re-admission, this reduction was determined to be insignificant. This contradicts current literature, which shows that multidisciplinary home care programs from other institutions have shown a significant reduction in length of stay between pre-enrolment and post-enrolment [13].

This could result from a culmination of factors that contribute to the length of stay in hospital, such as complicated family or social backgrounds or multiple medical co-morbidities [24,25]. We also postulate that with better patient education and communication between patients and healthcare providers, patients may have better health literacy and, in turn, better patient outcomes. In the event of a re-admission, the reason for a re-admission may be dire enough to warrant an equally long stay, leading to a non-significant decrease in the length of stay in the hospital.

With large effect size of reduction, the transitional care program benefited high utilizers the most. To reiterate, the program enrolled frequent admitters (more than twice in six months or more than thrice in a year), which utilized a high amount of hospital resources. Among them, we further selected high utilizers with three or more ED visits within three months in the pre-enrolment period, which translated to an average of at least one ED visit per month.

### 4.3. High Utilizers

Among the 391 high utilizers, the large effect size of reduction on ED visits showed that transitional care is very effective in reducing utilization among frequent ED attendees. In fact, this also suggested that the group that benefited the most from our transitional care program were the high utilizers.

Our results contradict what was found in one study that showed no difference in outcomes for more complex patients [26]. This could be due to the selection of matched controls in the study where a different methodology was used and how transitional care was delivered in each institution [20,27]. However, our results are consistent with other transitional care interventions that benefit high-need, high-cost patients [28,29].

### 4.4. Effect of COVID-19 on Outcome Variables

The transitional care program showed a similar reduction in ED re-attendances even during the COVID-19 pandemic (r = −0.7, *p* < 0.001). It was not affected significantly by COVID-19. Although there were movement restrictions and concerns about home visits from healthcare workers initially, rapid mitigation strategies shortened the periods of lockdown in Singapore and reduced the ability of the team to serve the patients during the pandemic [30]. The importance of transitional care programs was more greatly valued when access to specialist outpatient clinics was limited [31,32].

The second period was selected to analyze care outcomes when the transitional care program returned to normal operations or business as usual. The results concurred with the overall reduction in ED visits post-enrolment (r = −0.8, *p* < 0.001). The reason for this larger effect size achieved during the second period compared to the moderate effect size during the first period (COVID-19) could be due to the availability of a full spectrum of clinical care services.

### 4.5. Diagnoses Enrolled in Transitional Care Program

Our study also determined the three most common diagnoses of patients enrolled in the transitional care program to be fluid overload, urinary tract infection, and pneumonia. The main diagnosis for each enrolment was determined by the diagnosis of the most recent ED visit or inpatient admission. However, given the multiple co-morbidities of each patient, it is possible that each patient could have multiple reasons for admission [24,25]. Hence, it is difficult to determine the conditions that would benefit the most from the transitional care program. In addition, this list may merely reflect the more common reasons for hospital admission in general, which in turn became the use-case for this program itself [33,34].

### 4.6. Limitations

Our study has a few limitations. Firstly, our study is a single-arm study with no matched control. Secondly, re-admissions were measured within the same hospital, which did not include re-admissions or ED visits to other hospitals. Based on our experience, the vast majority of our patients are known to our community nurses and do not cross-visit to other hospitals due to geographic distance.

The cost-effectiveness of the program was out of the scope of this study and, therefore, not evaluated. Hence, future studies directed at a cost–benefit analysis of the program should be undertaken.

Another limitation is determining the main diagnosis of each enrolment, such as the diagnosis of the most recent ED visit or inpatient admission during the pre-enrolment period. As with any other retrospective study, there is a heavy dependency on the diagnosis coded by the medical officers. The hospital holds regular departmental meetings to emphasize accurate diagnosis coding. Despite good efforts, there may be instances of inaccurate diagnoses, leading to an under-representation of the patients’ actual condition or an incomplete picture of their health status, given that these diagnoses were likely based on a complex interplay of various medical and surgical conditions. As such, it is difficult to determine the types of conditions that would benefit most from the program.

## 5. Conclusions

This study validates and reinforces the effectiveness of transitional care programs in reducing hospital utilization while supplementing the lack of up-to-date literature on transitional care in Singapore and the Asia region. Not only were the total numbers of ED re-attendances reduced significantly, the number of frequent re-attendances also dropped significantly. This reduction was observed during COVID-19 and post-COVID periods, suggesting the viability of this model even in pandemics.

## Figures and Tables

**Table 1:** Components of SKH transitional care program (Hospital to Home) and the delivery.

Predischarge Components	Post Discharge Components
Discharge planning and needs assessment Family and caregiver training	Symptom management Medication reconciliation Functional assessment, home optimization Patient education and counseling Care coordination Multidisciplinary meeting Care transition

**Table 2:** Characteristics of the patients enrolled.

	n (%)
Total number of unique patients	1679
Age groups (years)	
20 to 40	16 (1)
41 to 60	124 (7.4)
61 to 80	856 (50.9)
>80	683 (40.7)
Gender	
Female	875 (52.1)
Male	804 (47.9)
Clinical Frailty Scale	n = 1328
0	0
1	0
2	2 (0.1)
3	23 (1.7)
4	204 (15.3)
5	353 (26.6)
6	399 (30.0)
7	333 (25.1)
8	14 (1.1)
9	0
Modified Barthel ADL index	n = 1229
0	89 (7.2)
1	64 (5.2)
2	27 (2.2)
3	45 (3.6)
4	52 (4.2)
5	36 (2.9)
6	38 (3.1)
7	32 (2.6)
8	36 (2.9)
9	40 (3.3)
10	45 (3.7)
11	42 (3.4)
12	68 (5.5)
13	64 (5.2)
14	43 (3.5)
15	47 (3.8)
16	45 (3.7)
17	64 (5.2)
18	63 (5.1)
19	72 (5.9)
20	217 (17.6)
Top ten diagnoses	n = 579
Fluid overload	148 (25.5)
Urinary tract infection	89 (15.4)
Pneumonia	83 (14.3)
Tendency to fall	49 (8.4)
Dizziness and giddiness	46 (7.9)
Chest pain	34 (5.9)
Unspecified injury of the head	34 (5.9)
Congestive heart failure	33 (5.7)
Dyspnea	32 (5.5)
Chronic obstructive pulmonary disease with acute exacerbation	31 (5.4)

## Data Availability

Data are contained within the article and Supplementary Materials.

## Supplementary Materials

The following supporting information can be downloaded at: https://www.mdpi.com/article/10.3390/healthcare12212144/s1, Table S1: Emergency Department (ED) visits across the study timeline; Table S2: Inpatient admissions across the study timeline; Table S3: Subgroup analysis of ED visits in high utilizers (n = 391).
