# Supplementary material for: Transitional Care Program in Reducing Acute Hospital Utilization in Singapore
Source: Healthcare (Basel). 2024 Oct 28;12(21):2144. doi: 10.3390/healthcare12212144 (PMC11545327; doi:10.3390/healthcare12212144)
Supplement: Supplementary file 1 [file healthcare-12-02144-s001.zip › healthcare-3234697-SI.pdf]

healthcare

MDPI

Supplementary Materials

Table S1. Emergency Department (ED) visits across the study timeline.

No. of ED visits	Pre-enrolment	Enrolment	Post-enrolment
0	83	913	1419
1	975	539	356
2	555	255	133
3	242	137	56
≥4	149	160	40
Total	2004	2004	2004

Table S2. Inpatient admissions across the study timeline.

No. of inpatient admissions	Pre-enrolment	Enrolment	Post-enrolment
0	230	1125	1549
1	957	445	289
2	499	210	98
3	206	98	43
≥4	112	126	25
Total	2004	2004	2004

Table S3. Subgroup analysis of ED visits in high utilizers (n = 391)

ED visit	Pre-enrolment	Enrolment	Post-enrolment
0		114	232
1		108	76
2		66	47
3	242	35	19
≥4	149	68	17
Total	1423	800	312
